# Integrated Genomic–Metabolomic Analysis for Tri-Categorical Classification of Type 2 Diabetes Status in the Korean Ansan–Ansung Cohort

**DOI:** 10.3390/ijms262311688

**Published:** 2025-12-02

**Authors:** Junho Cha, Sungkyoung Choi

**Affiliations:** 1 Department of Applied Artificial Intelligence, College of Computing, Hanyang University, 55 Hanyang-Daehak-ro, Sangnok-gu, Ansan 15588, Republic of Korea; chajunho822@hanyang.ac.kr; 2 Department of Mathematical Data Science, College of Computing, Hanyang University, 55 Hanyang-Daehak-ro, Sangnok-gu, Ansan 15588, Republic of Korea

**Keywords:** type 2 diabetes, integrated genomic–metabolomic analysis, tri-categorical classification, Korean genome and epidemiology study, baseline-category logistic regression

## Abstract

Identifying high-risk individuals for type 2 diabetes (T2D), particularly during prediabetes (PD), remains challenging owing to its complex metabolic etiology. In this study, we aimed to develop and validate an integrative multi-omics model for the tri-categorical classification of T2D status (Normal Glucose Tolerance, PD, and T2D) by combining genomic and metabolomic data from a Korean cohort. Based on cross-sectional data from 1819 participants in the Ansan–Ansung cohort, significant metabolites associated with glycemic traits and T2D status were identified using regression analysis. A metabolite-adjusted genome-wide association study (GWAS) was conducted to identify T2D-associated genetic variants. Finally, three nested prediction models (Clinical, Metabolite-Enriched, and integrated Multi-omics) were constructed using baseline-category logistic regression and evaluated using stratified five-fold cross-validation. Thirty-nine metabolites were identified as consistently associated with T2D status and related glycemic traits. GWAS identified 86 T2D-associated independent single-nucleotide polymorphisms (SNPs). The final integrated multi-omics model, combining clinical factors, 39 metabolites, and 86 SNPs, demonstrated strong predictive performance for classifying T2D status, achieving an area under the receiver operating characteristic curve (AUC) of 0.935, significantly improved over the clinical model (AUC = 0.695) and metabolite-enriched model (AUC = 0.874). It also outperformed previously established external models and represents an important step in our understanding of T2D status. Our findings thus demonstrate that integrating genomic and metabolomic data provides a useful framework for the tri-categorical classification of T2D status. This multi-omics approach significantly enhances risk stratification beyond that provided by clinical or single-omics data alone, thus offering valuable insights into the underlying pathophysiology in T2D with potential for shaping future T2D research and clinical practice.

## 1. Introduction

Type 2 diabetes (T2D) is one of the most pressing metabolic disorders of the 21st century, and its prevalence continues to increase across diverse populations [1,2]. The 2021 report from the International Diabetes Federation revealed that diabetes affects more than half a billion people worldwide, a number projected to surge to 783 million by 2045 [2,3]. T2D, a complex metabolic disorder characterized by hyperglycemia, significantly increases the risk of severe complications, including cardiovascular disease (CVD), nephropathy, and retinopathy [4,5,6]. Even individuals in the intermediate stage of impaired glucose regulation, known as prediabetes (PD), face a significantly increased risk of progression to overt diabetes and its complications [7]. The substantial burden of T2D and PD on public health underscores the urgent need for improved strategies for early detection, prevention, and treatment to mitigate the risk of these debilitating comorbidities [8].

Genome-wide association studies (GWASs) have significantly advanced our understanding of the genetic basis of T2D by successfully identifying numerous T2D-associated variants and related glycemic traits [9,10,11]. These large-scale genetic studies have provided valuable insights into the hereditary components of T2D. Nevertheless, a considerable gap remains in translating these genetic discoveries into a clear understanding of the underlying biological mechanisms [12]. A primary challenge is that the identified genetic loci often account for only a small portion of the disease heritability, and their direct functional impact on T2D development remains unclear [13,14]. Further, most GWAS have focused on dichotomous comparisons (e.g., T2D vs. non-T2D), which may overlook the metabolic continuum from normoglycemia to PD and eventually to overt diabetes. Thus, translating the GWAS findings into clinical applications remains a major challenge.

Metabolomics has emerged as a powerful approach to bridge this gap and complement the insights from genomics [15]. This field involves a comprehensive analysis of small molecules (metabolites) and offers a real-time snapshot of the physiological state of an individual, reflecting the intricate interplay between genetic predispositions and environmental influences [16]. A key advantage of this approach is its ability to detect the distinct metabolic changes that occur before the clinical onset of hyperglycemia. Thus, metabolomic profiling is a valuable strategy for early T2D detection and identification of the molecular pathways that drive the disease [17,18]. Nevertheless, it is essential to acknowledge that confounding factors such as diet and lifestyle can affect metabolomic data [19]. Furthermore, in observational studies, determining whether the observed metabolic alterations are a cause or consequence of the disease process can be challenging [20].

To overcome the inherent limitations of single-omics approaches, we employed an integrative strategy combining genomic and metabolomic data [21]. This approach has the potential to significantly enhance our understanding of T2D pathophysiology by linking genetic risk factors with their corresponding functional metabolic outcomes [22]. By leveraging the complementary strengths of genomics and metabolomics, we aimed to identify more robust and reliable biomarkers to improve the prediction of T2D status. A key innovation of our study is the development of a predictive model that classifies individuals into three distinct categories: normal glucose tolerance (NGT), PD, and T2D. This tri-categorical classification moves beyond traditional binary models, aiming to provide a more precise and clinically relevant assessment of individual risk and to gain deeper insights into the complex pathophysiology underlying glucose dysregulation.

## 2. Results

We conducted a stepwise analysis to (i) identify metabolite signals linked to glycemic dysregulation, (ii) nominate genomic variants associated with tri-categorical T2D status, and (iii) quantify the incremental predictive value of integrating metabolites and single-nucleotide polymorphisms (SNPs) into clinical models. Unless otherwise noted, all models were adjusted for age, sex, body mass index (BMI), and high-density lipoprotein (HDL) cholesterol and adhered to the analysis plan described in the Section 4.

### 2.1. Characteristics of the Study Population

The final analytical cohort comprised 1819 participants from the Ansan–Ansung study, who were categorized into the following three groups: NGT (*n* = 747), PD (*n* = 736), and T2D (*n* = 336). Demographic and clinical characteristics of the study population, stratified based on T2D status, are shown in Table 1.

A comparative analysis of the groups revealed a statistically significant trend of worsening metabolic health across T2D status categories. Participants in the PD (mean age 57.47 ± 8.90 years) and T2D (56.60 ± 8.64 years) groups were significantly older than those in the NGT (54.94 ± 8.56 years) groups. A similar trend was observed for obesity, with the mean BMI increasing from 23.59 ± 2.96 kg/m^2^ in the NGT group to 25.35 ± 2.99 kg/m^2^ in the T2D group.

This pattern of metabolic decline was also evident in the lipid profiles. A distinct dyslipidemia pattern was observed, characterized by a significant increase in total cholesterol (TCHL) levels (from 186.80 ± 32.55 mg/dL in NGT to 201.31 ± 35.31 mg/dL in T2D) and a corresponding decrease in protective HDL cholesterol levels (from 45.37 ± 10.22 mg/dL in NGT to 42.07 ± 9.93 mg/dL in T2D).

As mandated by the diagnostic criteria, all glycemic indices demonstrated marked and significant deterioration across the groups. For instance, mean Glycated or Glycosylated Hemoglobin A1c (HbA1c) increased from 5.23 ± 0.24% in the NGT group to 6.38 ± 0.98% in the T2D group. Similarly, Homeostasis Model Assessment of Insulin Resistance (HOMA-IR), an insulin resistance marker, escalated from 1.42 ± 0.65 (NGT) to 2.38 ± 1.26 (T2D). These baseline comparisons confirmed the substantial burden of established metabolic risk factors in the T2D group, validating the clinical relevance of this stratification.

To further dissect T2D-related characteristics and identify common significant metabolites, we conducted two additional subgroup analyses. First, the participants were re-stratified into three groups based on their HbA1c levels; the results are detailed in Table S1. In this classification, nearly all variables, including age, BMI, and lipid profiles, differed significantly across the groups (*p*-value < 0.0001), except sex (*p*-value = 0.158, chi-squared test). Second, to capture more granular intermediate phenotypes, participants were classified into six subgroups based on combinations of Isolated Impaired Fasting Glucose (IFG) and Isolated Impaired Glucose Tolerance (IGT) (Table S2). This analysis revealed significant differences in most characteristics (*p*-value < 0.0001), except in low-density lipoprotein (LDL) cholesterol, which did not differ significantly between the more detailed subgroups (*p*-value = 0.139, ANOVA).

### 2.2. Metabolites Associated with Glycemic Traits and T2D Status

To identify a robust set of metabolites associated with glucose dysregulation, we used a multifaceted analytical strategy. Regression analyses were performed on both continuous glycemic traits (fasting glucose levels (FPG), 2 h postprandial plasma glucose levels (2h-PG), HbA1c, and HOMA-IR) and several categorical T2D classification schemes (T2D status according to American Diabetes Association (ADA) criteria or HbA1c level and six subgroups).

Using multivariable linear regression, we identified 63, 74, 61, and 62 metabolites as being significantly associated (*q*-value < 0.05) with FPG, 2h-PG, HbA1c, and HOMA-IR, respectively. In parallel, we utilized baseline-category logistic regression to analyze categorical T2D classification schemes. This analysis identified 89 significant metabolites using the ADA criteria, 72 using the HbA1c-based classification, and 102 when participants were stratified into six glucose subgroups (NGT, IFG, IGT, IFG+IGT, IFG+T2D, and T2D).

**Figure 1:**
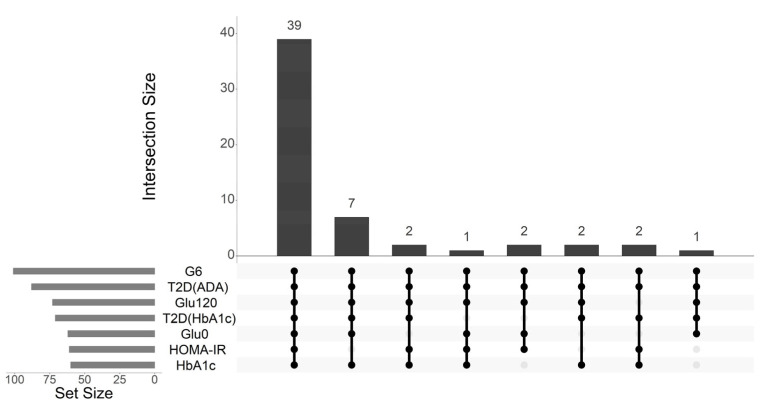
Upset plot showing metabolites consistently identified as significant.

In summary, we identified metabolites that were consistently significant across all seven analytical frameworks (four continuous and three categorical). This intersectional analysis, visualized as an Upset plot (Figure 1), yielded a final set of 39 key metabolites. This panel of selected biomarkers, used for all subsequent integrative modeling, comprises six amino acids, seven sphingolipids, twenty-five glycerophospholipids, and one hexose. The detailed statistical results for these 39 metabolites across all analyses are presented in Table 2.

### 2.3. Genetic Variant Associated with T2D Status

To investigate the genetic underpinnings of T2D status, we conducted a GWAS using the tri-categorical T2D status as the primary phenotype. This analysis was comprehensively adjusted for clinical covariates and 39 previously identified metabolites to identify genetic signals independent of major metabolic shifts.

The analysis initially identified a pool of 113 unique SNPs that were significantly associated (*p*-value < 1 *×* 10^−4^) with at least one disease transition. Particularly, 64 SNPs were associated with the transition from NGT to PD (Figure S1), and 50 SNPs were associated with the transition from NGT to T2D (Figure S2). Only one SNP, *rs1878618*, was significantly associated with both transitions.

To address potential redundancy due to Linkage Disequilibrium (LD), we implemented a pruning procedure. For any group of SNPs exhibiting high LD (*r*^2^ > 0.8), only the variant with the lowest *p*-value was retained for subsequent analyses. This filtering step refined the initial list to a set of independent genetic markers. After LD pruning, 39 SNPs remained for the NGT vs. PD comparison, and 48 SNPs remained for the NGT vs. T2D comparison.

Considering the single overlapping SNP, a final set of 86 unique genetic variants (*p*-value < 1 *×* 10^−4^) was used to construct an integrated prediction model. A complete list of these 86 SNPs, including their association statistics and annotations, is provided in Table S3.

### 2.4. Performance of T2D Prediction Models

The predictive capabilities of T2D status were evaluated in the Ansan–Ansung cohort by constructing a series of three nested baseline-category logit models. The performances of these models, assessed using a comprehensive suite of metrics, including the area under the receiver operating characteristic (ROC) curve (AUC), area under the precision–recall curve (AUPRC), and others, calculated through micro-averaging, are detailed in Table 3.

The clinical risk model, relying solely on standard clinical variables, established a baseline AUC of 0.695 and an AUPRC of 0.523. The integration of 39 significant metabolites in the metabolite-enriched model substantially enhanced the predictive power, with the AUC and AUPRC increasing to 0.874 and 0.768, respectively. The final integrated multi-omics model, which combined clinical variables, 39 metabolites, and 86 selected SNPs, achieved the best performance. This comprehensive model yielded a final AUC of 0.935 and an AUPRC of 0.879, demonstrating a clear and substantial improvement in T2D status prediction with the addition of metabolomic and genomic information.

### 2.5. Comparison with Existing Prediction Models

**Figure 2:**
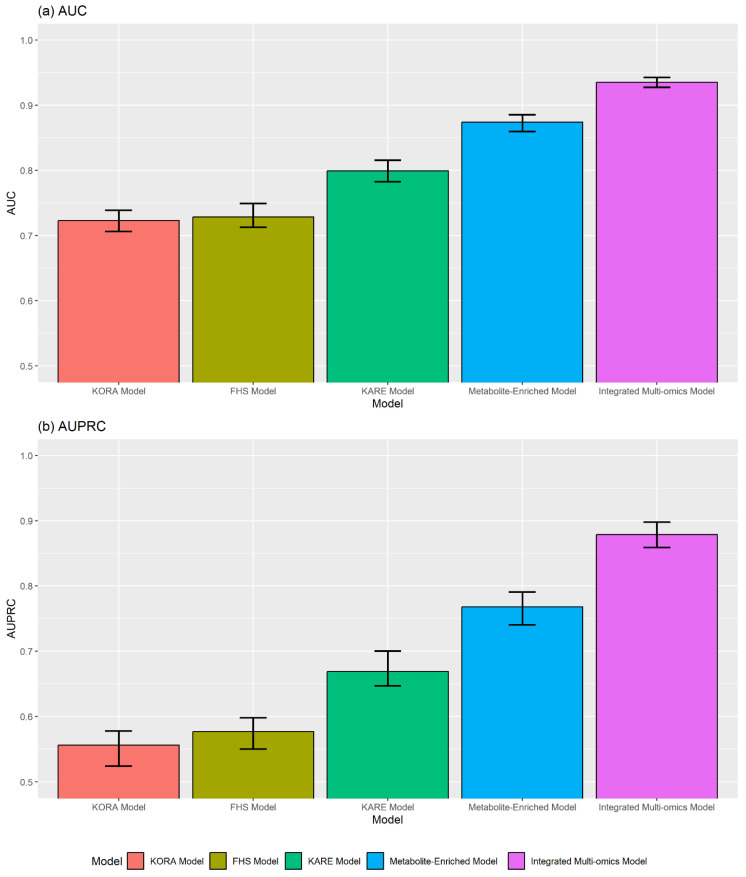
Comparative performance of T2D status classification models. (**a**) the area under the receiver operating characteristic curve (AUC); (**b**) the area under the precision–recall curve (AUPRC).

In a subsequent analysis, we benchmarked the performance of our newly developed models against several previously published metabolite-based prediction models, including those from the Framing Heart Study (FHS) [35], Cooperative Health Research in the Region of Augsburg (KORA) [46], and Korean Association REsource (KARE) [57] studies. We applied these external models to our Ansan–Ansung cohort dataset to facilitate direct comparison; the results are summarized in Figure 2.

Our metabolite-enriched model (AUC: 0.874, 95% confidence interval [CI]: 0.860–0.885) significantly outperformed all three previously established models: the KORA (AUC: 0.723, 95% CI: 0.703–0.744), FHS (AUC: 0.728, 95% CI: 0.711–0.745), and KARE (AUC: 0.799, CI: 0.784–0.814). A similar pattern of superiority was observed for the AUPRC metrics. Our model achieved an AUPRC of 0.768 (95% CI: 0740–0.790), which was substantially higher than that of the KORA (0.556, 95% CI: 0.524–0.578), FHS (0.577, 95% CI: 0.550–0.598), and KARE (0.669, 95% CI: 0.647–0.700) models.

Furthermore, a comparison between our own models underscored the significance of adding genetic information. Our integrated multi-omics model (AUC: 0.935, 95% CI: 0.928–0.943; AUPRC: 0.879, 95% CI: 0.859–0.898) not only outperformed all external models but also demonstrated a statistically significant improvement over our metabolite-enriched model across both AUC and AUPRC metrics. This result confirmed that integrating the selected genetic markers substantially enhanced the ability to accurately classify individuals across the T2D status spectrum.

### 2.6. Functional Annotation of Associated Genetic Variants

A functional annotation study was conducted on the 86 SNPs identified through the GWAS to assess their potential biological relevance and pathogenic risk. Each variant was annotated using the NCBI dbSNP database (hg19/GRCh37), with intergenic SNPs mapped to the nearest upstream or downstream gene.

We used a suite of in silico tools to evaluate their potential pathogenicity. Variants were scored using Combined Annotation-Dependent Depletion (CADD) [58], Deleterious Annotation of genetic variants using Neural Networks (DANN) [59], and RegulomeDB [60] databases. Based on the established threshold, defined as having a CADD score ≥ 12.37, DANN score ≥ 0.8, or RegulomeDB score < 3, a total of 23 SNPs were classified as having a high-risk score.

We then cross-referenced the 82 genes corresponding to the 86 SNPs against the literature for previously reported associations with diabetes-related phenotypes (including T2D, obesity, and CVD). This review revealed that 36 of these genes had been previously implicated in relevant pathologies (Table S4). Among this set of 36 genes, 10 were linked to SNPs that met our high-risk score criteria, strengthening the evidence for their involvement in T2D.

Among the identified variants, *rs1704349* is a known expression quantitative trait locus (eQTL) that exhibits a RegulomeDB score of 1f, indicating strong evidence for its location within a regulatory region. Moreover, the *PLA2G4E* gene in its locus has been previously associated with both obesity [61] and CVD [62]. This combination of regulatory evidence and functional context highlights *rs1704349* as a key variant for further investigation into the genetic mechanisms underlying T2D.

## 3. Discussion

In this cross-sectional study of a Korean community-based cohort, we demonstrate that integrating clinical, metabolic, and genomic data significantly enhances the tri-categorical classification of T2D status (NGT, PD, and T2D). Our findings demonstrate the effectiveness of a multi-omics approach in accurately classifying individuals along the spectrum of glucose dysregulation and highlight its superiority over traditional clinical risk and previously established metabolite-based models. This significant advancement in T2D classification methods is a testament to the potential of multi-omics approaches.

Our research demonstrates the successful application of an integrative analytical framework to tri-categorical disease outcomes. Moving beyond a simple binary classification, our model provides a more nuanced prediction that reflects the progressive nature of T2D. This is particularly relevant for identifying individuals in the prediabetic stage, which is a critical window for clinical intervention. Our final integrated multi-omics model, combining clinical factors with a panel of 39 metabolites and 86 genetic variants, achieved strong predictive performance, with an AUC of 0.935. This result strongly supports the hypothesis that integrating data across multiple biological layers provides a more comprehensive and accurate picture of an individual’s disease risk.

The 39-metabolite signature identified in our study not only serves as a powerful predictive tool but also provides significant insights into the underlying pathophysiology of T2D. Among the 39 selected metabolites (Table 2), the effects of the primary metabolites on T2D were as follows:

### 3.1. Valine

**Figure 3:**
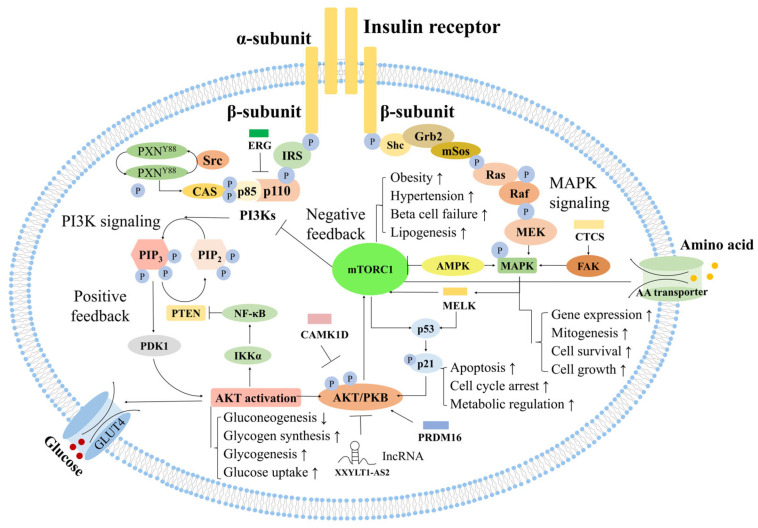
Schematic diagram of the PI3K–AKT–mTORC1 signaling pathway and its role in insulin resistance. The upward and downward arrows denote increased activation and inhibition of the pathway components, respectively.

For several years, elevated circulating levels of valine, a branched-chain amino acid (BCAA), have been reported as a characteristic of amino acid dysregulation in T2D [42,43,44]. Recent studies have reported that the amino acid valine (Table 2) regulates essential physiological processes, such as glucose metabolism and cell growth, by activating mTORC1, the rapamycin complex 1 [44,63,64,65] (Figure 3). The level of valine also influences various physiological processes, including glycolysis, inflammation, energy metabolism, and mitochondrial biogenesis [43,44]. Further, increased valine concentrations contribute to fatty acid accumulation in skeletal muscles and are associated with insulin resistance [40,41].

### 3.2. Alanine

In the liver, L-alanine (Table 2) transfers its amino group to alpha-ketoglutarate via the liver enzyme alanine aminotransferase (ALT) to form pyruvate, which is then converted to glucose [23,25]. Therefore, high ALT and alanine levels can lead to excessive glucose production and are reported to be closely related to T2D development in several previous studies [24,26]. In T2D, the glucose–alanine cycle increases owing to insulin resistance and excessive inflammatory free fatty acids, thus increasing alanine levels [66].

### 3.3. Glutamate and Glutamine

Glutamate (Table 2) is abundant in various foods and promotes insulin secretion in pancreatic β-cells as a metabolic coupling factor [67]. In healthy people, because of its wide distribution in the human body, glutamate concentration in the blood is low owing to rapid oxidation in the small intestine after ingestion. However, in patients with insulin resistance, such as those with T2D and obesity, amino acid and protein metabolism are abnormal [33,68,69,70,71], and plasma glutamate levels are significantly elevated [31,32,33,72]. In this case, platelet activation caused by increased oxidative stress and inflammation may lead to hyperglutamatemia [73,74,75,76]. This is likely to increase pancreatic β-cell susceptibility [77,78] and activate glutamate receptors in β-cells, leading to β-cell dysfunction and apoptosis [34]. Glutamine, the precursor of glutamate (Table 2), activates mTORC1 [28] (Figure 3) and is involved in regulating pancreatic β-cell function and insulin secretion [27]. In addition, it plays a vital role in regulating α-cell mass in animal models [29,79]. Additionally, glutamine and glutamate are closely related to energy metabolism, and their ratio can reflect the overall state of energy metabolism [30]. An imbalance between glutamine and glutamate is hypothesized to inhibit α-cell proliferation and thus limit β-cell regeneration [30].

### 3.4. Glycine

Glycine (Table 2) is an important component of proteins that serves as a substrate for metabolic processes and participates in regulating important biological functions, including the synthesis of purine, heme, creatinine, and glutathione [38,80,81]. Unlike BCAA compounds such as valine, glycine is normally synthesized in vivo and is additionally replenished and maintained through food intake [38,39,81,82]. Nonetheless, low plasma glycine levels are reported to increase the risk of metabolic diseases, such as T2D, non-alcoholic fatty liver disease (NAFLD), hypertension, metabolic syndrome, obesity, inflammation, and oxidative stress [17,37,38,39]. Decreased glycine levels have already been identified as T2D-related metabolic biomarkers in many previous diabetes-related studies [35,36,46,57]. Glycine depletion may accelerate T2D by exposing pancreatic cells to oxidative stress [66,83].

### 3.5. Lysophosphatidylcholine (lysoPC) Acyl C18:2

The lysophospholipid family, which includes lysoPC, lysophosphatidylserine, and lysophosphatidic acid, has diverse physiological and pathological functions as bioactive lipid mediators [84]. Particularly, lysoPC participates in various metabolic processes, including cell proliferation, inflammation, and glucose metabolism [85,86]. Previous studies have reported that lysoPC acyl C18:2 is present at lower concentrations in patients with diabetes than in the normal group [18,46]. In a β-cell-derived cell line, the lysophospholipid family increases glucose-dependent insulin secretion through an orphan G-protein-coupled receptor; thus, reduced lysoPC acyl C18:2 (Table S3) levels could potentially lead to impaired insulin secretion [45].

### 3.6. Hexose

In general, elevated blood hexose levels are the most prominent feature of T2D [54,56]. Excessive consumption of Hexose (Table S3) is known to affect blood glucose levels, leading to insulin resistance and reduced insulin sensitivity [52,53,55]. In skeletal muscle cells, glucose is absorbed via glucose transporter 4 (GLUT4), and when insulin signaling is disrupted, such as by AKT, PI3K, and mTORC1, blood glucose levels increase because of insulin resistance [52,53,55]. Therefore, increased blood glucose levels are an important biomarker of T2D.

### 3.7. Sphingomyelins

Metabolic disturbances in blood lipids, such as cholesterol, sphingolipids, and triglycerides (TGs), are associated with the development of T2D [48,87]. Insulin affects the enzymes involved in lipid metabolism in the adipose tissue and liver. T2D and insulin resistance can alter plasma lipid levels, which can serve as potential biomarkers for diabetes [88,89,90]. In particular, changes in plasma sphingolipid concentration and composition are features of diabetes [47,91,92] and have been reported as potential predictors of diabetes [47,91,92,93,94,95,96]. Disruption of sphingolipid metabolism has been linked to inflammation regulation, insulin secretion, β-cell function, and β-cell death [48,97,98,99,100,101]. Among these, sphingomyelin is the most abundant sphingolipid in plasma, accounting for approximately 85–90% of the major sphingolipids and is associated with T1D, T2D, coronary artery disease, and obesity [49,50,51]. Therefore, sphingomyelins, such as C16:0, C16:1, C18:1, and C24:1 (Table S3), may serve as potential prognostic biomarkers for diabetes and its complications, reflecting insulin secretion and β-cell function.

Central to these insights is the PI3K–AKT–mTORC1 signaling pathway (Figure 3), a critical regulator of cellular metabolism and insulin action [44,63,64,65,102,103,104]. In carbohydrate metabolism, particularly in skeletal muscles, which absorb more than 50% of glucose, insulin signaling is crucial for postprandial blood sugar control [105,106]. Protein kinase B (PKB), known as AKT, is activated by phosphoinositide 3-kinases (PI3K) and mitogen-activated protein kinase (MAPK) signaling pathways, which are the key regulatory mechanisms of insulin signaling, and glucose is absorbed from the blood through the GLUT4 receptor [107,108,109,110]. Our biomarker panel reflects the systematic metabolic dysregulation deeply intertwined with this pathway.

The identified amino acids are key indicators of this disruption. For instance, increased levels of BCAAs (valine and alanine) are well-established contributors to insulin resistance through mTORC1 overactivation, which initiates a negative feedback loop that impairs insulin receptor substrate-1 (IRS-1) signaling [40,66,111]. Similarly, alterations in glutamate and glutamine levels are indicative of disrupted energy metabolism [30]. This metabolic dysregulation is understood to be both a cause and consequence of aberrant mTORC1 signaling, a pathway also deeply involved in regulating pancreatic β-cell function and insulin secretion [27]. Furthermore, the lipid species in our biomarker panel, including lysoPC acyl C18:2 and various sphingomyelins, converge in these pathological processes. Lipid dysregulation can lead to the accumulation of intermediates such as ceramides, which impair AKT activation and promote inflammation, thereby exacerbating insulin resistance [48,84,87,112,113]. The robustness of this 39-metabolite panel, validated by its superior performance compared to biomarker sets from the FHS, KORA, and KARE studies, underscores its clinical utility as a direct reflection of these core metabolic disturbances.

Our metabolite-adjusted GWAS successfully identified 86 independent SNPs associated with T2D status, further enriching our understanding. Functional annotation of these variants provided valuable biological context, as exemplified by *rs1704349*, a candidate variant with high regulatory potential linked to the cardiometabolically relevant gene *PLA2G4E* [61,62]. The fact that 36 of the implicated genes have been previously linked to diabetes-related phenotypes further validates our genetic findings, as detailed in Table S4. These genetic variants offer valuable insights into the genetic basis of T2D and may be used for risk prediction and personalized medicine.

A key consideration of our model is its potential for clinical translation. We acknowledge that a full multi-omics panel is expensive for routine screening. However, our results demonstrate the strong performance of the metabolite-enriched model (Model 2; AUC = 0.874). This model, which combines clinical factors with a 39-metabolite panel, represents a more feasible and cost-effective approach for identifying high-risk individuals. Therefore, the value of genomic data (Model 3) lies not only in the increased predictive accuracy but also in its ability to identify novel targets, such as *PLA2G4E*, for future therapeutic development.

Despite the strengths of this study, several limitations must be acknowledged when contextualizing our findings. First, the cross-sectional design of the analysis, using data from a single follow-up point, prevented us from establishing causal relationships or tracking the temporal dynamics of the identified biomarkers in relation to disease onset. Second, this study was conducted exclusively in a Korean cohort. Although this enhances the relevance of our findings for this specific population, genetic architecture and metabolic profiles can differ across ethnicities. Therefore, the generalizability of our model to other ethnic groups requires further investigation. Third, although our classification model demonstrated high accuracy, our analysis relied on internal validation (stratified cross-validation [CV]). We were unable to validate these findings in an independent external dataset because of the lack of a cohort comparable to both the Korean Chip genomic and p180 metabolomic data. Because biomarker selection and model evaluation were conducted within the same cohort, the reported predictive performance may be somewhat optimistic and should be interpreted cautiously until validated in independent datasets. Fourth, our metabolite-adjusted GWAS approach, which is helpful in identifying independent predictors, may introduce collider bias and is not intended for the general biological discovery of all T2D loci. Finally, although we adjusted for major confounding factors, the potential for residual confounding from unmeasured variables such as dietary and lifestyle factors cannot be entirely ruled out. This residual confounding factor could have affected the accuracy of our predictive model and should be considered in future studies.

## 4. Materials and Methods

### 4.1. Study Cohort and Design

In this study, we utilized data derived from the Ansan and Ansung cohort, part of the Korean Genome and Epidemiology Study (KoGES), a long-term community-based prospective cohort initiated in 2001–2002 [114]. The primary objective of the KoGES was to assess the genetic and environmental risk factors for chronic diseases in the Korean population. The well-characterized Ansan (urban) and Ansung (rural) sub-cohorts were included in the study. At the baseline (2001–2002), 10,030 individuals aged 40–69 years were recruited, and the participants underwent repeated follow-up examinations every 2 years. For this analysis, we used a dataset from the second follow-up survey conducted between 2005 and 2006, which included both genomic and metabolomic data. All data were provided by the Genome Center of the Korea National Institute of Health (KNIH). Comprehensive, standardized quality control (QC) of the genomic and metabolomic data has already been performed at the institutional level by the KNIH before distribution, ensuring a high-quality dataset for analysis.

The dataset available from the second follow-up survey initially included 5583 individuals. To ensure the quality of our analysis, we applied the predefined exclusion criteria. Among the initially included individuals, those with missing genotypes or metabolite profiles, incomplete clinical data, or prior baseline diagnoses of diabetes were excluded. We also excluded individuals receiving oral hypoglycemic drugs or insulin therapy, as well as those with major chronic diseases such as cancer or cardiovascular events, because such conditions may confound metabolic measurements [115,116]. After applying these filtering processes, a total of 1819 participants were included in the study. Ethical considerations of this study were reviewed by the Institutional Review Board of Hanyang University (IRB no. HYUIRB-202402-010). All participants provided written informed consent prior to their inclusion in the study.

### 4.2. Assessment of Clinical and Biochemical Factors

In each survey, participants completed a standardized questionnaire that covered demographic information, lifestyle factors (including smoking, alcohol intake, and physical activity), and medical history. Anthropometric measurements, including weight, height, waist circumference, and BMI, were performed by trained staff. Blood pressure was measured in a seated position after a period of rest.

Biochemical parameters were assessed after overnight fasting. FPG, 2h-PG, and HbA1c levels were measured according to standardized procedures. Lipid profiles, including TG, TCHL, LDL cholesterol, HDL cholesterol, and fasting insulin (INSO) levels, were also measured. Insulin resistance was estimated using the HOMA_IR, calculated as follows: FPG (mg/dL) × INSO (μU/mL)/405 [117].

### 4.3. Definition of T2D Status

Participants were stratified into three categories—NGT, PD, or T2D—according to the ADA diagnostic criteria [118,119]. The specific thresholds for this classification were as follows: NGT was defined as HbA1c < 5.7%, FPG < 100 mg/dL, and 2h-PG < 140 mg/dL; PD was defined as 5.7% ≤ HbA1c ≤ 6.4%, 100 mg/dL ≤ FPG ≤ 125 mg/dL, or 140 mg/dL ≤ 2h-PG ≤ 199 mg/dL; T2D was defined as HbA1c ≥ 6.5%, FPG ≥ 126 mg/dL, 2h-PG ≥ 200 mg/dL, or a self-reported physician’s diagnosis with anti-diabetic medication.

### 4.4. Genotyping and QC

Genotyping was performed using the Korean Chip (KORV1.1), an array specifically designed for the Korean population, which provides a high coverage of common variants relevant to this ancestry. The Korean chip interrogated approximately 830,000 SNPs across the genome [120].

A data QC procedure was applied to the raw genotyping data using PLINK v1.9.0 software (National Institutes of Health, Bethesda, MD, USA) [121]. The QC process was performed as follows: Participants were excluded based on a high missing rate (>5%), extreme heterozygosity, and discrepancies between genetic and reported sex; SNPs were removed if they exhibited a high missing call rate (>5%), a minor allele frequency (MAF) of less than 5%, or a significant deviation from the Hardy–Weinberg equilibrium (HWE) at a *p*-value threshold of 1.0 × 10^−5^. After rigorous QC, a total of 546,591 SNPs and 1819 individuals were included in the analysis. Because our primary goal was predictive accuracy using established markers rather than novel locus discovery, we did not apply imputation to the genomic data.

### 4.5. Metabolomic Profiling and Preprocessing

Targeted metabolomic profiling of plasma samples was performed using AbsoluteIDQ™ p180 Kit (Biocrates Life Sciences AG, Innsbruck, Austria) coupled with a mass spectrometry platform. This kit enabled simultaneous quantification of 186 metabolites across six major classes: 40 acylcarnitines, 19 biogenic amines, 21 amino acids, 15 sphingolipids, 90 glycerophospholipids, and a hexose.

Metabolites with >20% missing values or values below the limit of detection were excluded. For reproducibility, all detailed QC, normalization, and preprocessing steps were performed following established protocols from a previous study using this cohort [122]. After QC, 121 metabolites were retained for further analysis. These metabolites were selected based on their known or suspected roles in metabolic pathways associated with T2D, including glucose metabolism, lipid metabolism, and insulin sensitivity. The concentrations were log-transformed and standardized (using z-scores) to minimize skewness and facilitate comparisons across features.

### 4.6. Statistical Analyses

#### 4.6.1. Selection of Metabolites Linked to Glycemic Traits

**Figure 4:**
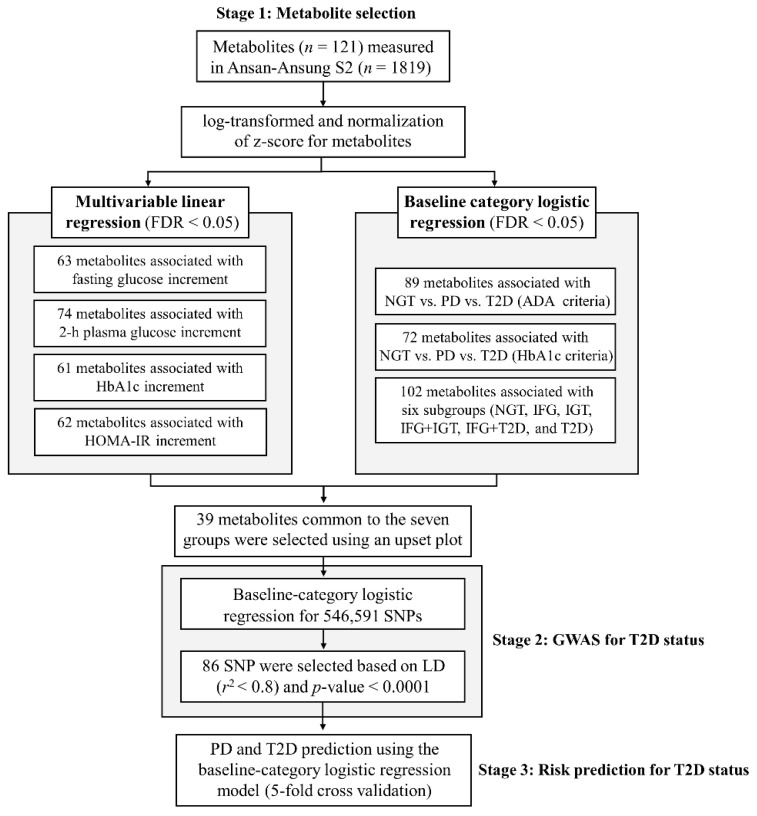
Flowchart of multi-omics analysis. The diagram outlines the overall study design, including participant selection, classification into T2D status (Normal Glucose Tolerance [NGT], Prediabetes [PD], and T2D), acquisition of genomic and metabolomic data, statistical association testing, integrative modeling, and predictive performance evaluation.

To comprehensively evaluate the relationship between metabolite profiles and glycemic regulation, we first analyzed the continuous phenotypes related to T2D (Figure 4). The following four quantitative traits were considered: FPG, 2h-PG, HbA1c, and HOMA-IR. Associations between metabolite concentrations and continuous variables were evaluated using a multivariate linear regression model adjusted for age, sex, BMI, and HDL cholesterol.

In parallel, analyses using a categorical subgroup definition to capture the distinct stages of glucose dysregulation were performed. A total of 1819 participants were classified according to the following scheme:

1. ADA-based T2D status: Participants were grouped into NGT (=1), PD (=2), or T2D (=3) groups according to the ADA criteria.
2. HbA1c-based grouping: Based on HbA1c levels, participants were classified into the following three groups: normal (<5.7%), PD (5.7–6.4%), and T2D (≥6.5%).
3. Six glucose subgroups: To further delineate intermediate phenotypes, we defined six categories based on the FPG and 2h-PG levels. NGT was defined as FPG < 100 mg/dL and 2h-PG < 140 mg/dL. IFG was defined as 100 ≤ FPG ≤ 125 mg/dL with 2h-PG < 140 mg/dL, whereas IGT was defined as FPG < 100 mg/dL with 140 ≤ 2h-PG < 200 mg/dL. Combined IFG and IGT was defined as 100 ≤ FPG ≤ 125 mg/dL and 140 ≤ 2h-PG < 200 mg/dL. Further, we specified an IFG with diabetes-range 2h-PG (IFG+T2D) group (100 ≤ FPG ≤ 125 mg/dL and 2h-PG ≥ 200 mg/dL) and a T2D with normal FPG group (FPG < 100 mg/dL and 2h-PG ≥ 200 mg/dL).

For these categorical outcomes, baseline-category logistic regression models were applied using the same covariates as those in the linear regression models. This dual strategy allowed us to assess whether metabolite associations were consistent across continuous variation and discrete diagnostic classifications.

Metabolites demonstrating significant associations in either framework were subjected to false discovery rate (FDR) correction (*q*-value < 0.05) to account for multiple testing. Only those that passed the FDR threshold were used as candidate biomarkers after integrating the genomic and metabolomic analyses.

#### 4.6.2. Genomic Association with T2D Status

Building on these metabolomic findings, we identified genetic variants associated with T2D status. A GWAS was conducted using genomic data from 1819 individuals in the Ansan–Ansung cohort, incorporating 546,591 SNPs that met the QC criteria.

The core of our strategy was a baseline-category logistic regression model designed to test the association of each genetic variant with T2D status (NGT, PD, and T2D) as defined by the ADA criteria. A critical feature of our statistical approach was the comprehensive adjustment for covariates. In addition to accounting for standard demographic, clinical, and lipid profiles (age, sex, BMI, and HDL cholesterol level), the model was uniquely adjusted for the entire set of metabolites identified in the previous stage. The rationale for this adjustment was to specifically identify genetic markers with an independent contribution to T2D status for their value in the integrated prediction model.

Rather than employing a stringent genome-wide discovery threshold (typically, *p*-value < 5 × 10^−8^), which is the standard for novel locus discovery, we adopted a more moderate *p*-value cutoff (*p*-value < 1 × 10^−4^) to identify the candidate variants for inclusion in our subsequent predictive modeling. To avoid redundancy because of LD, we pruned the nominated SNPs to retain only those with an LD *r*^2^ value of less than 0.8, effectively creating a set of largely independent genetic predictors. The resulting collection of pruned SNPs constituted the final set of genetic factors to be integrated into comprehensive risk prediction models.

#### 4.6.3. Construction and Assessment of Prediction Models

In the final phase of our analysis, we systematically constructed and rigorously evaluated a series of predictive models for T2D. Our primary objective was to assess the ability of previous approaches to discriminate between three diagnostic categories, NGT, PD, and T2D, and thereby assess the added value of integrating multiple omics.

We developed a series of three nested models, each building on the last, using baseline-category logistic regression as the core algorithm:

1. Model 1 (Clinical Risk Model): This foundational model included only standard demographic, clinical, and lipid variables (age, sex, BMI, and HDL cholesterol level) to establish baseline predictive performance.
2. Model 2 (Metabolite-Enriched Model): The second model was augmented by incorporating the full panel of metabolites identified as significant in our initial association analyses. This model was designed to assess the predictive contributions of the metabolic signatures.
3. Model 3 (Integrated Multi-omics Model): The final model integrated the set of pruned independent SNPs from the GWAS alongside clinical and metabolomic factors. This model represents the full-scale multi-omics approach.

We must clarify our methodological distinction between explanatory feature selection (Section 4.6.1 and Section 4.6.2) and predictive modeling. Feature selection was performed on the full dataset using statistical associations (*p*-values) to identify robust explanatory biomarkers rather than to optimize predictive accuracy. This preselected set of 125 features (39 metabolites and 86 SNPs) was used as the fixed input for the predictive modeling phase.

The predictive performance of each model was assessed using a stratified five-fold CV strategy. This approach was specifically chosen to account for the imbalance in the T2D status (NGT, PD, and T2D). The procedure involved partitioning the dataset into five subsets or folds while maintaining the same class proportions in each fold as in the original dataset. In each of the five iterations, one fold was used as the test set, whereas the remaining four were used for model training. The performance metrics were then averaged across all five folds to produce a single stable estimate of predictive performance.

To ensure robust assessment, we evaluated the performance of each model for multiple metrics. Discriminative ability was primarily assessed using the AUC and AUPRC. To gain a more detailed understanding of model performance, we also calculated the accuracy (ACC), sensitivity, specificity, precision, Matthews correlation coefficient (MCC), F1-score (F1), and balanced accuracy (BA). Given the multiclass nature of our variables, all performance metrics were calculated for each class using a one-vs.-rest (OvR) approach.

#### 4.6.4. Functional Annotation of the Associated Loci

To translate the GWAS statistical associations into biological insights, a comprehensive functional annotation of the identified genetic loci was performed. The primary objectives of this analysis were to elucidate the functional consequences of the top-associated SNPs, map them to candidate causal genes, and place these findings within the context of established biological pathways.

We employed a stepwise annotation framework to prioritize variants systematically:

1. Initial mapping to genes and regulatory regions: We conducted variant annotation using ANNOVAR (version 2018Apr16, https://annovar.openbioinformatics.org/en/latest/ (accessed on 26 November 2025)) with the hg19/GRCh37 reference genome and Ensemble gene build 106 [123]. All SNPs that exceeded the prespecified significance threshold were first mapped to their nearest genes and subsequently interrogated using additional publicly available resources, including the Ensembl VEP and Regulome DB databases.
2. Assessment of deleteriousness and pathogenicity: Each variant was further evaluated using CADD [58] and DANN [59] scores. These complementary tools integrate evolutionary conservation, biochemical features, and regulatory context to estimate the probability of a variant being functionally deleterious. Variants exceeding commonly applied thresholds (CADD ≥ 12.37 or DANN ≥ 0.8) were prioritized for downstream consideration.
3. Integration with expression and regulatory databases: To gain insight into potential issue-specific effects, prioritized variants were cross-referenced with eQTL datasets and RegulomeDB annotations [60]. Particular attention was given to variants with a RegulomeDB score of 3 or lower, as these are more likely to lie within transcription factor-binding sites or DNase-hypersensitive regions with regulatory potential. This step enabled the identification of variants associated with altered gene expression in metabolically relevant tissues such as the liver, adipose tissue, and pancreas.

This functional annotation phase complemented the predictive modeling analyses by providing mechanistic insights into the molecular pathways through which the identified variants may contribute to T2D onset and status.

## 5. Conclusions

In conclusion, we demonstrate that an integrative multi-omics approach combining clinical, genomic, and metabolomic data is a powerful and highly accurate tool for the tri-categorical classification of T2D status. The developed model significantly improves risk stratification beyond that possible using clinical or single-omics data alone, and its superiority is confirmed through a direct comparison with previously established models. The specific metabolites and genetic variants identified serve as robust biomarkers and offer valuable insights into the underlying pathophysiology of T2D, particularly highlighting the central role of the PI3K–AKT–mTORC1 signaling pathway. These findings pave the way for more personalized risk assessments and targeted preventive strategies in the future, particularly for identifying individuals at the critical prediabetic stage.

## Figures and Tables

**Table 1:** Demographic and clinical characteristics of the study population.

	Type 2 Diabetes (T2D) Status Based on the American Diabetes Association (ADA) Criteria			*p*-Value
	Normal Glucose Tolerance (NGT) (*n* = 747)	Prediabetes (PD) (*n* = 736)	T2D (*n* = 336)	
SEX				<0.0001
Male	316 (42.30%)	357 (48.51%)	204 (60.71%)	
Female	431 (57.70%)	379 (51.49%)	132 (39.29%)	
AGE (years) ^a^	54.94 ± 8.56	57.47 ± 8.90	56.60 ± 8.64	<0.0001
BMI (kg/m^2^) ^b^	23.59 ± 2.96	25.14 ± 3.21	25.35 ± 2.99	<0.0001
HDL ^c^	45.37 ± 10.22	43.02 ± 9.51	42.07 ± 9.93	<0.0001
LDL ^d^	118.73 ± 30.10	122.61 ± 34.55	123.35 ± 35.63	0.031
TG ^e^	113.51 ± 64.55	161.89 ± 144.66	179.47 ± 131.38	<0.0001
HbA1c ^f^	5.23 ± 0.24	5.72 ± 0.32	6.38 ± 0.98	<0.0001
FPG ^g^	83.74 ± 5.32	97.47 ± 9.84	115.82 ± 29.18	<0.0001
2h-PG ^h^	93.79 ± 18.32	138.99 ± 33.95	238.50 ± 58.22	<0.0001
INSO ^i^	6.82 ± 2.97	8.20 ± 3.59	8.20 ± 3.62	<0.0001
TCHL ^j^	186.80 ± 32.55	198.01 ± 36.20	201.31 ± 35.31	<0.0001
HOMA_IR ^k^	1.42 ± 0.65	1.99 ± 0.93	2.38 ± 1.26	<0.0001

^a^ Means ± standard deviation (SD); ^b^ body mass index; ^c^ high-density lipoprotein; ^d^ low-density lipoprotein; ^e^ triglyceride; ^f^ glycated hemoglobin levels; ^g^ fasting glucose levels; ^h^ 2 h postprandial plasma glucose levels; ^i^ fasting insulin; ^j^ total cholesterol; ^k^ homeostasis model assessment of insulin resistance.

**Table 2:** Detailed results for the 39 selected metabolites across seven type 2 diabetes (T2D)-related traits.

Metabolite	Linear Regression Beta				Baseline-Category Logistic Regression Odds Ratio (OR)—95% Confidence Interval (CI)									Ref.
	Glu0	Glu120	HbA1c	HOMA-IR	Criteria by ADA		Criteria by HbA1c		Six Subgroups (G6)					
					PD	T2D	PD	T2D	IFG	IGT	IFG+IGT	IFG+T2D	T2D	
Alanine	3.546	11.084	0.104	0.183	1.623 (1.44–1.83)	1.742 (1.50–2.02)	1.432 (1.28–1.60)	1.690 (1.370–2.08)	1.733 (1.46–2.07)	1.338 (1.15–1.56)	1.641 (1.36–1.98)	1.704 (1.38–2.11)	1.790 (1.49–2.15)	[23,24,25,26]
Glutamine	−2.895	−5.019	−0.065	−0.078	1.181 (1.04–1.34)	0.825 (0.73–0.94)	1.152 (1.02–1.30)	0.723 (0.62–0.85)	0.958 (0.81–1.13)	1.312 (1.10–1.56)	0.885 (0.75–1.05)	0.869 (0.72–1.04)	0.809 (0.70–0.94)	[27,28,29,30]
Glutamate	2.025	7.377	0.082	0.112	1.317 (1.17–1.48)	1.492 (1.30–1.72)	1.169 (1.05–1.31)	1.477 (1.23–1.77)	1.283 (1.09–1.51)	1.065 (0.91–1.25)	1.130 (0.94–1.35)	1.199 (0.98–1.47)	1.544 (1.32–1.81)	[31,32,33,34]
Glycine	−3.179	−13.062	−0.096	−0.107	0.771 (0.69–0.86)	0.490 (0.42–0.58)	0.802 (0.72–0.90)	0.579 (0.47–0.72)	0.728 (0.61–0.87)	0.747 (0.64–0.87)	0.677 (0.56–0.82)	0.493 (0.39–0.62)	0.488 (0.40–0.60)	[35,36,37,38,39]
Proline	1.312	3.750	0.039	0.124	1.371 (1.22–1.54)	1.228 (1.06–1.42)	1.247 (1.12–1.39)	1.179 (0.97–1.44)	1.372 (1.17–1.62)	1.345 (1.16–1.56)	1.236 (1.03–1.48)	1.161 (0.95–1.43)	1.218 (1.02–1.45)	-
Valine	2.069	10.079	0.089	0.137	1.593 (1.41–1.80)	1.504 (1.29–1.75)	1.412 (1.26–1.59)	1.446 (1.17–1.79)	1.317 (1.10–1.58)	1.503 (1.28–1.76)	1.359 (1.12–1.65)	1.223 (0.98–1.52)	1.604 (1.33–1.94)	[40,41,42,43,44]
Lysophosphatidylcholine acyl C17:0	−1.337	−9.621	−0.085	−0.070	0.887 (0.80–0.99)	0.615 (0.53–0.71)	0.841 (0.76–0.94)	0.645 (0.53–0.79)	1.063 (0.91–1.25)	0.814 (0.70–0.94)	0.874 (0.74–1.04)	0.593 (0.48–0.73)	0.668 (0.56–0.80)	-
Lysophosphatidylcholine acyl C18:2	−2.244	−13.128	−0.087	−0.082	0.768 (0.68–0.87)	0.552 (0.47–0.64)	0.787 (0.70–0.88)	0.574 (0.47–0.71)	0.946 (0.79–1.13)	0.748 (0.64–0.88)	0.601 (0.50–0.73)	0.586 (0.47–0.73)	0.564 (0.47–0.68)	[18,45,46]
Phosphatidylcholine diacyl C30:2	1.106	3.578	0.063	0.113	1.029 (0.92–1.15)	1.269 (1.11–1.45)	1.148 (1.03–1.28)	1.495 (1.25–1.79)	1.049 (0.89–1.24)	0.970 (0.83–1.13)	0.974 (0.82–1.16)	1.073 (0.88–1.31)	1.392 (1.19–1.63)	-
Phosphatidylcholine diacyl C32:1	1.238	5.771	0.083	0.123	1.187 (1.06–1.33)	1.423 (1.24–1.64)	1.288 (1.16–1.44)	1.534 (1.28–1.85)	1.118 (0.95–1.32)	1.215 (1.05–1.41)	1.153 (0.97–1.38)	1.170 (0.96–1.43)	1.542 (1.31–1.82)	-
Phosphatidylcholine diacyl C34:1	2.302	8.975	0.110	0.139	1.376 (1.23–1.55)	1.771 (1.53–2.05)	1.476 (1.32–1.65)	1.819 (1.51–2.20)	1.349 (1.14–1.60)	1.295 (1.11–1.51)	1.305 (1.09–1.56)	1.436 (1.17–1.76)	1.957 (1.65–2.32)	-
Phosphatidylcholine diacyl C34:2	2.364	8.378	0.112	0.139	1.256 (1.12–1.41)	1.749 (1.51–2.02)	1.364 (1.22–1.52)	1.873 (1.55–2.27)	1.449 (1.22–1.71)	1.199 (1.03–1.39)	1.089 (0.91–1.30)	1.526 (1.25–1.87)	1.838 (1.54–2.19)	-
Phosphatidylcholine diacyl C34:4	2.406	4.157	0.076	0.127	1.331 (1.19–1.49)	1.359 (1.18–1.57)	1.241 (1.11–1.38)	1.357 (1.13–1.64)	1.585 (1.35–1.86)	1.116 (0.96–1.30)	1.396 (1.17–1.66)	1.156 (0.94–1.42)	1.409 (1.19–1.67)	-
Phosphatidylcholine diacyl C36:1	1.574	5.092	0.085	0.131	1.363 (1.22–1.53)	1.473 (1.28–1.70)	1.411 (1.27–1.57)	1.526 (1.27–1.84)	1.339 (1.14–1.58)	1.308 (1.13–1.51)	1.196 (1.00–1.43)	1.241 (1.02–1.52)	1.558 (1.32–1.84)	-
Phosphatidylcholine diacyl C36:2	1.666	3.662	0.072	0.125	1.231 (1.10–1.38)	1.445 (1.26–1.66)	1.266 (1.14–1.41)	1.479 (1.23–1.78)	1.498 (1.27–1.77)	1.156 (1.00–1.34)	1.041 (0.87–1.24)	1.302 (1.07–1.59)	1.484 (1.25–1.76)	-
Phosphatidylcholine diacyl C36:4	2.392	5.926	0.080	0.108	1.229 (1.10–1.38)	1.457 (1.26–1.68)	1.185 (1.06–1.32)	1.477 (1.22–1.79)	1.393 (1.18–1.65)	0.999 (0.86–1.16)	1.253 (1.05–1.50)	1.266 (1.03–1.55)	1.567 (1.32–1.86)	-
Phosphatidylcholine diacyl C36:5	3.313	9.998	0.083	0.152	1.456 (1.30–1.63)	1.726 (1.49–2.00)	1.221 (1.10–1.36)	1.568 (1.28–1.92)	1.639 (1.38–1.94)	1.178 (1.01–1.37)	1.642 (1.37–1.97)	1.538 (1.25–1.89)	1.911 (1.59–2.30)	-
Phosphatidylcholine diacyl C38:5	2.789	7.325	0.073	0.138	1.421 (1.27–1.59)	1.589 (1.38–1.83)	1.206 (1.08–1.34)	1.473 (1.21–1.79)	1.587 (1.34–1.88)	1.129 (0.97–1.31)	1.522 (1.27–1.82)	1.396 (1.14–1.71)	1.716 (1.44–2.05)	-
Phosphatidylcholine diacyl C38:6	2.493	11.166	0.068	0.100	1.314 (1.17–1.47)	1.723 (1.49–2.00)	1.231 (1.10–1.37)	1.563 (1.28–1.91)	1.349 (1.14–1.60)	1.236 (1.06–1.44)	1.349 (1.13–1.62)	1.579 (1.28–1.95)	2.066 (1.72–2.48)	-
Phosphatidylcholine diacyl C40:5	1.570	5.209	0.063	0.101	1.336 (1.19–1.49)	1.360 (1.18–1.57)	1.260 (1.13–1.40)	1.378 (1.14–1.67)	1.278 (1.08–1.51)	1.201 (1.04–1.39)	1.386 (1.16–1.66)	1.205 (0.98–1.48)	1.444 (1.22–1.72)	-
Phosphatidylcholine diacyl C40:6	1.273	7.090	0.038	0.088	1.212 (1.09–1.35)	1.398 (1.22–1.67)	1.189 (1.07–1.32)	1.287 (1.06–1.56)	1.152 (0.98–1.36)	1.240 (1.07–1.44)	1.167 (0.98–1.39)	1.387 (1.13–1.70)	1.532 (1.29–1.82)	-
Phosphatidylcholine diacyl C42:0	−1.917	−7.155	−0.074	−0.055	0.644 (0.57–0.73)	0.666 (0.57–0.77)	0.701 (0.62–0.79)	0.761 (0.61–0.94)	0.669 (0.56–0.80)	0.614 (0.52–0.73)	0.705 (0.58–0.85)	0.623 (0.50–0.78)	0.791 (0.66–0.95)	-
Phosphatidylcholine diacyl C42:1	−1.989	−7.005	−0.077	−0.076	0.657 (0.58–0.74)	0.675 (0.58–0.79)	0.725 (0.65–0.81)	0.763 (0.61–0.95)	0.676 (0.57–0.81)	0.650 (0.55–0.77)	0.687 (0.57–0.83)	0.660 (0.53–0.83)	0.763 (0.63–0.92)	-
Phosphatidylcholine diacyl C42:5	2.007	5.350	0.039	0.130	1.202 (1.07–1.35)	1.399 (1.22–1.60)	1.065 (0.96–1.18)	1.364 (1.15–1.62)	1.312 (1.12–1.53)	1.067 (0.92–1.25)	1.277 (1.08–1.51)	1.313 (1.09–1.59)	1.491 (1.28–1.74)	-
Phosphatidylcholine acyl–alkyl C34:3	−3.095	−11.585	−0.066	−0.103	0.646 (0.57–0.74)	0.624 (0.53–0.74)	0.813 (0.72–0.92)	0.832 (0.67–1.04)	0.692 (0.57–0.84)	0.656 (0.55–0.78)	0.477 (0.38–0.59)	0.596 (0.47–0.76)	0.670 (0.55–0.82)	-
Phosphatidylcholine acyl–alkyl C40:3	−1.369	−4.267	−0.038	−0.061	0.728 (0.65–0.82)	0.881 (0.76–1.02)	0.840 (0.75–0.94)	0.995 (0.81–1.22)	0.761 (0.64–0.91)	0.671 (0.57–0.79)	0.732 (0.60–0.89)	0.800 (0.65–0.99)	0.975 (0.82–1.16)	-
Phosphatidylcholine acyl–alkyl C40:5	2.229	5.218	0.041	0.089	1.219 (1.08–1.37)	1.384 (1.19–1.61)	1.143 (1.02–1.28)	1.284 (1.05–1.58)	1.457 (1.22–1.74)	0.950 (0.81–1.12)	1.355 (1.12–1.63)	1.248 (1.01–1.55)	1.618 (1.35–1.94)	-
Phosphatidylcholine acyl–alkyl C42:1	−2.046	−9.762	−0.076	−0.095	0.687 (0.61–0.78)	0.671 (0.57–0.78)	0.734 (0.65–0.83)	0.746 (0.60–0.93)	0.759 (0.64–0.91)	0.652 (0.55–0.77)	0.664 (0.54–0.81)	0.635 (0.50–0.80)	0.711 (0.59–0.86)	-
Phosphatidylcholine acyl–alkyl C42:4	−2.318	−8.109	−0.057	−0.098	0.648 (0.58–0.73)	0.658 (0.57–0.77)	0.774 (0.69–0.87)	0.844 (0.68–1.04)	0.632 (0.53–0.76)	0.613 (0.52–0.73)	0.657 (0.54–0.80)	0.616 (0.49–0.77)	0.741 (0.62–0.89)	-
Phosphatidylcholine acyl–alkyl C44:4	−2.876	−8.058	−0.075	−0.101	0.667 (0.59–0.75)	0.618 (0.53–0.72)	0.747 (0.67–0.84)	0.757 (0.61–0.94)	0.580 (0.48–0.70)	0.690 (0.59–0.81)	0.651 (0.53–0.79)	0.541 (0.43–0.68)	0.713 (0.59–0.86)	-
Phosphatidylcholine acyl–alkyl C44:6	−2.425	−9.002	−0.078	−0.082	0.606 (0.54–0.68)	0.587 (0.50–0.69)	0.691 (0.62–0.78)	0.728 (0.59–0.91)	0.590 (0.49–0.71)	0.603 (0.51–0.71)	0.652 (0.54–0.79)	0.522 (0.42–0.66)	0.681 (0.57–0.82)	-
Hydroxysphingomyeline C14:1	−2.901	−7.242	−0.068	−0.100	0.727 (0.65–0.82)	0.645 (0.56–0.75)	0.887 (0.79–0.99)	0.772 (0.63–0.95)	0.649 (0.55–0.77)	0.800 (0.68–0.94)	0.630 (0.52–0.76)	0.636 (0.51–0.79)	0.690 (0.57–0.83)	-
Hydroxysphingomyeline C16:1	−2.607	−7.032	−0.062	−0.084	0.777 (0.69–0.87)	0.636 (0.55–0.74)	0.950 (0.85–1.06)	0.764 (0.62–0.94)	0.676 (0.57–0.81)	0.841 (0.72–0.99)	0.682 (0.57–0.82)	0.627 (0.51–0.77)	0.687 (0.57–0.82)	-
Hydroxysphingomyeline C22:2	−4.245	−12.596	−0.109	−0.159	0.599 (0.52–0.69)	0.451 (0.38–0.54)	0.758 (0.67–0.86)	0.579 (0.46–0.73)	0.489 (0.40–0.60)	0.649 (0.54–0.78)	0.501 (0.41–0.62)	0.510 (0.40–0.65)	0.442 (0.36–0.54)	-
Sphingomyeline C16:0	−2.944	−10.466	−0.056	−0.127	0.671 (0.59–0.76)	0.567 (0.49–0.66)	0.862 (0.77–0.97)	0.822 (0.67–1.01)	0.611 (0.51–0.73)	0.652 (0.55–0.77)	0.532 (0.44–0.65)	0.505 (0.41–0.63)	0.623 (0.52–0.75)	[47,48,49,50,51]
Sphingomyeline C16:1	−3.564	−10.665	−0.068	−0.127	0.685 (0.60–0.78)	0.5560 (0.47–0.66)	0.865 (0.77–0.98)	0.759 (0.61–0.95)	0.582 (0.48–0.71)	0.712 (0.60–0.85)	0.519 (0.42–0.64)	0.532 (0.42–0.68)	0.578 (0.47–0.71)	-
Sphingomyeline C18:1	−2.891	−4.906	−0.043	−0.091	0.784 (0.69–0.89)	0.640 (0.55–0.75)	0.966 (0.86–1.09)	0.769 (0.62–0.95)	0.561 (0.47–0.68)	0.881 (0.75–1.04)	0.663 (0.54–0.81)	0.688 (0.55–0.86)	0.672 (0.55–0.82)	-
Sphingomyeline C24:1	−2.231	−7.499	−0.069	−0.095	0.693 (0.62–0.78)	0.647 (0.56–0.75)	0.805 (0.72–0.90)	0.749 (0.62–0.91)	0.662 (0.56–0.79)	0.616 (0.53–0.72)	0.639 (0.53–0.77)	0.690 (0.56–0.85)	0.676 (0.57–0.81)	-
Hexose	13.101	32.126	0.338	0.360	1.916 (1.64–2.24)	7.566 (6.04–9.48)	1.694 (1.48–1.94)	7.469 (5.73–9.74)	3.728 (2.96–4.70)	1.133 (0.93–1.39)	3.893 (3.06–4.96)	8.413 (6.47–10.95)	8.576 (6.70–10.98)	[52,53,54,55,56]

**Table 3:** Performance evaluation of T2D status classification models.

Model	AUC ^a^	AUPRC ^b^	Sensitivity ^c^	Specificity ^d^	Precision ^e^	ACC ^f^	MCC ^g^	F1 ^h^	BA ^i^
Clinical Risk Model	0.695	0.523	0.712	0.488	0.410	0.563	0.192	0.521	0.600
Metabolite-Enriched Model	0.874	0.768	0.825	0.749	0.622	0.774	0.545	0.709	0.787
Integrated Multi-omics Model	0.935	0.879	0.888	0.525	0.483	0.646	0.400	0.626	0.707

^a^ Area under the ROC curve (AUC); ^b^ area under the precision–recall Curve (AUPRC); ^c^ true positive rate (sensitivity); ^d^ true negative rate (specificity); ^e^ positive predictive values (precision); ^f^ accuracy (ACC); ^g^ Matthews correlation coefficient (MCC); ^h^ F1-score (F1), the harmonic mean of precision and recall; ^i^ balanced accuracy (BA), the average between sensitivity and specificity.

## Data Availability

The datasets presented in this article are not readily available because they contain human genomic and metabolomic information that is accessible only through an approval process regulated by the Korea National Institute of Health (https://is.kdca.go.kr/ (accessed on 10 March 2025)). Requests to access the datasets should be directed to the Korea National Institute of Health genome data request portal (https://is.kdca.go.kr/).

## Supplementary Materials

The following supporting information can be downloaded at: https://www.mdpi.com/article/10.3390/ijms262311688/s1. References [124,125,126,127,128,129,130,131,132,133,134,135,136,137,138,139,140,141,142,143,144,145,146,147,148,149,150,151,152,153,154,155,156,157,158,159,160,161,162,163,164,165,166,167,168,169,170,171,172,173,174,175,176,177,178,179,180,181,182,183,184,185,186,187,188,189,190,191,192,193,194,195,196,197,198,199,200,201,202,203,204,205,206,207,208,209,210,211,212,213,214,215,216,217,218,219,220,221,222,223,224,225,226,227,228,229] are cited in Supplementary Materials.

## Abbreviations

The following abbreviations are used in this manuscript:

ACC	Accuracy
ADA	American Diabetes Association
AUC	Area Under the Receiver Operating Characteristic Curve
AUPRC	Area Under the Precision–Recall Curve
BA	Balanced Accuracy
BMI	Body Mass Index
CADD	Combined Annotation-Dependent Depletion
CV	Cross-Validation
CVD	Cardiovascular Disease
DANN	Deleterious Annotation of genetic variants using Neural Networks
PD	Prediabetes
GWAS	Genome-Wide Association Study
SNP	Single-Nucleotide Polymorphisms
eQTL	Expression Quantitative Trait Locus
F1	F1-Score
FDR	False Discovery Rate
FHS	Framingham Heart Study
FPG	Fasting Plasma Glucose
HbA1c	Glycated Hemoglobin
HOMA-IR	Homeostasis Model Assessment of Insulin Resistance
HDL	High-Density Lipoprotein Cholesterol
HWE	Hardy–Weinberg Equilibrium
IFG	Isolated Impaired Fasting Glucose
IGT	Impaired Glucose Tolerance
KARE	Korean Association Resource
KoGES	Korean Genome and Epidemiology Study
KORA	Cooperative Health Research in the Region of Augsburg
LD	Linkage Disequilibrium
LDL	Low-Density Lipoprotein Cholesterol
MAPK	Mitogen-Activated Protein Kinase
MCC	Matthews Correlation Coefficient
mTORC1	Mammalian target of rapamycin complex 1
NGT	Normal Glucose Tolerance
OvR	One-vs.-Rest
TG	Triglyceride
TCHL	Total Cholesterol
PI3K	Phosphoinositide 3-Kinase
QC	Quality Control
ROC	Receiver Operating Characteristic
SNP	Single-Nucleotide Polymorphism
T2D	Type 2 Diabetes
2h-PG	2 h postprandial Plasma Glucose levels
