# Supplementary material for: Integrated Genomic–Metabolomic Analysis for Tri-Categorical Classification of Type 2 Diabetes Status in the Korean Ansan–Ansung Cohort
Source: Int J Mol Sci. 2025 Dec 2;26(23):11688. doi: 10.3390/ijms262311688 (PMC12691764; doi:10.3390/ijms262311688)
Supplement: Supplementary file 1 [file ijms-26-11688-s001.zip › ijms-3960179-supplementary.pdf]

Supplementary Tables and Figures

Integrated Genomic–Metabolomic Analysis for Tri-Categorical Classification of Type 2 Diabetes Status in the Korean Ansan–Ansung Cohort

Junho Cha [1] and Sungkyoung Choi [1,2,*]

[1] Department of Applied Artificial Intelligence, College of Computing, Hanyang University, 55 Hanyang-Daehak-ro, Sangnok-gu, Ansan 15588, South Korea; chajunho822@hanyang.ac.kr
[2] Department of Mathematical Data Science, College of Computing, Hanyang University, 55 Hanyang-Daehak-ro, Sangnok-gu, Ansan 15588, South Korea; day0413@hanyang.ac.kr
* Correspondence: day0413@hanyang.ac.kr; Tel.: +82-31-400-5465

Table S1. Baseline characteristics of the study population stratified by HbA1c levels.

	T2D Status by HbA1c levels			*P*-value
	**G1_HbA1c (*n* = 1,058)**	**G2_HbA1c (*n* = 632)**	**G3_HbA1c (*n* = 129)**	
**SEX**				0.158
**Male**	518 (48.96%)	289 (45.73%)	70 (54.26%)	
**Female**	540 (51.04%)	379 (51.49%)	132 (39.29%)	
**AGE(years)** [a]	55.13 ± 8.74	57.99 ± 8.55	57.26 ± 8.89	< 0.0001
**BMI(kg/m^2)** [b]	23.90 ± 3.02	25.36 ± 3.18	25.85 ± 2.97	< 0.0001
**HDL** [c]	45.20 ± 10.64	42.41 ± 8.60	39.33 ± 8.11	< 0.0001
**LDL** [d]	117.78 ± 32.41	125.94 ± 32.24	125.33 ± 38.69	< 0.0001
**TG** [e]	128.65 ± 116.46	160.22 ± 101.09	208.34 ± 174.33	< 0.0001
**INS0** [f]	7.13 ± 3.08	8.30 ± 3.71	8.48 ± 3.88	< 0.0001
**TCHL** [g]	188.71 ± 33.72	200.39 ± 35.31	206.33 ± 37.18	< 0.0001
**HOMA_IR** [h]	1.58 ± 0.76	2.04 ± 1.01	2.79 ± 1.42	< 0.0001
**FPG**	88.61 ± 10.23	98.45 ± 12.68	133.56 ± 36.22	< 0.0001
**2h-PG**	111.92 ± 41.21	158.73 ± 47.99	261.74 ± 82.55	< 0.0001

[a] Means ± standard deviation (SD); [b] body mass index; [c] high-density lipoprotein; [d] low-density lipoprotein; [e] triglyceride, [f] fasting Insulin; [g] total cholesterol; [h] homeostasis model assessment of insulin resistance.

Table S2. Baseline characteristics of the study population stratified by six glucose subgroups.

	Six glucose subgroups by the combination of IFG and IGT						
	**NGT (*n* = 894)**	**IFG [a] (*n* = 202)**	**IGT [b] (*n* = 252)**	**IFG+IGT (*n* = 173)**	**IFG+T2D (*n* = 123)**	**T2D (*n* = 175)**	***P*-value**
**SEX**							
**Male**	360 (40.27%)	141 (69.80%)	90 (35.71%)	101 (58.38%)	77 (62.60%)	108 (61.71%)	
**Female**	534 (59.73%)	61 (30.20%)	162 (64.29%)	72 (41.62%)	46 (37.40%)	67 (38.29%)	
**AGE(years)**	55.90 ± 8.81	55.69 ± 8.56	59.35 ± 8.85	54.47 ± 8.20	56.63 ± 8.43	55.95 ± 8.70	< 0.0001
**BMI(kg/m^2)**	23.72 ± 3.01	25.15 ± 3.02	25.11 ± 3.37	26.00 ± 3.03	25.69 ± 2.73	24.97 ± 3.08	< 0.0001
**HDL**	44.91 ± 9.99	43.66 ± 10.50	42.71 ± 9.03	42.63 ± 9.69	42.27 ± 9.72	42.20 ± 10.42	< 0.0001
**LDL**	119.89 ± 30.81	118.77 ± 37.51	125.24 ± 31.64	120.05 ± 36.68	122.98 ± 31.77	124.26 ± 37.24	0.139
**TG**	118.36 ± 70.74	173.43 ± 202.37	158.69 ± 93.46	174.43 ± 159.65	165.32 ± 85.36	187.98 ± 153.24	< 0.0001
**INS0**	6.87 ± 2.93	8.47 ± 3.78	8.10 ± 3.46	8.96 ± 3.84	8.22 ± 3.72	8.14 ± 3.72	< 0.0001
**TCHL**	188.47 ± 33.56	197.12 ± 38.18	199.69 ± 35.81	197.57 ± 33.00	198.31 ± 33.73	204.05 ± 36.67	< 0.0001
**HOMA_IR**	1.44 ± 0.65	2.20 ± 0.98	1.85 ± 0.83	2.37 ± 1.04	2.27 ± 1.09	2.45 ± 1.43	< 0.0001
**FPG**	84.65 ± 6.25	105.39 ± 5.09	92.07 ± 10.56	106.62 ± 5.53	111.12 ± 6.84	119.59 ± 38.84	< 0.0001
**2h-PG**	96.21 ± 20.41	109.47 ± 21.68	163.02 ± 16.24	167.68 ± 17.68	238.27 ± 30.46	256.98 ± 60.53	< 0.0001

[a] Impaired fasting glucose (IFG); [b] impaired glucose tolerance (IGT).

Table S3. 86 significant SNPs were identified from the metabolite-adjusted GWAS.

Chr [a]	BP [b]	rsID [c]	MAF [d]	Ref [e]	Alt [f]	OR [g]	95% CI [h]	STAT	*P*-value
Significant 39 SNPs in NGT vs PD									
15	65875380	*rs8042910*	0.035	G	C	0.293	0.172-0.500	-4.502	6.74×10^{-6}
6	31199573	*rs11967600*	0.149	C	T	1.867	1.414-2.465	4.404	1.06×10^{-5}
14	102310529	*rs116924463*	0.036	G	A	0.306	0.18-0.522	-4.354	1.33×10^{-5}
18	2007871	*rs1940653*	0.230	G	A	0.595	0.471-0.752	-4.342	1.41×10^{-5}
11	8334841	*rs7102266*	0.333	C	A	1.575	1.280-1.938	4.298	1.73×10^{-5}
8	79875221	*rs12682560*	0.219	T	C	0.606	0.481-0.762	-4.270	1.95×10^{-5}
14	83755467	*rs76610020*	0.062	C	T	2.401	1.606-3.591	4.268	1.97×10^{-5}
4	133933873	*rs114228107*	0.017	C	A	0.155	0.066-0.366	-4.260	2.05×10^{-5}
15	58794965	*rs143940303*	0.032	G	A	3.375	1.916-5.945	4.212	2.53×10^{-5}
19	13696046	*rs72620558*	0.473	T	C	0.660	0.543-0.801	-4.206	2.60×10^{-5}
1	2991808	*rs2993482*	0.203	T	G	0.604	0.477-0.765	-4.184	2.86×10^{-5}
20	31764158	*rs78326465*	0.013	G	A	10.088	3.397-29.964	4.163	3.14×10^{-5}
8	36921711	*rs143491218*	0.038	G	A	3.072	1.797-5.253	4.102	4.10×10^{-5}
1	82787818	*rs1857814*	0.307	G	A	0.655	0.534-0.803	-4.074	4.61×10^{-5}
2	175201299	*rs62174227*	0.373	G	C	1.513	1.239-1.848	4.069	4.72×10^{-5}
3	100869763	*rs76693940*	0.100	A	G	1.964	1.418-2.72	4.064	4.83×10^{-5}
2	223674830	*rs62187185*	0.034	C	T	3.389	1.878-6.115	4.055	5.02×10^{-5}
10	12579459	*rs10458822*	0.363	A	G	0.660	0.54-0.807	-4.054	5.04×10^{-5}
6	170261237	*rs714189*	0.065	T	C	2.271	1.527-3.38	4.047	5.18×10^{-5}
15	38355543	*rs12591780*	0.434	G	A	1.492	1.228-1.812	4.031	5.54×10^{-5}
4	17429859	*rs77606380*	0.060	G	T	0.424	0.279-0.645	-4.007	6.14×10^{-5}
4	127931569	*rs145233789*	0.020	A	C	0.227	0.110-0.470	-4.004	6.24×10^{-5}
17	17090099	*rs77103270*	0.165	A	G	1.697	1.308-2.203	3.979	6.91×10^{-5}
2	18110269	*rs11675993*	0.066	T	C	2.281	1.518-3.429	3.967	7.29×10^{-5}
15	65916527	*rs3743171*	0.338	T	A	0.660	0.537-0.810	-3.964	7.38×10^{-5}
10	125678755	*rs1878618*	0.282	A	G	1.542	1.244-1.910	3.957	7.58×10^{-5}
15	42276359	*rs1704349*	0.031	G	A	0.313	0.176-0.558	-3.950	7.81×10^{-5}
8	146288293	*rs76088403*	0.251	C	T	1.568	1.254-1.961	3.946	7.95×10^{-5}
1	193441154	*rs4539115*	0.070	A	G	0.452	0.304-0.671	-3.941	8.11×10^{-5}
1	57867333	*rs77859272*	0.011	C	T	9.595	3.109-29.613	3.934	8.35×10^{-5}
18	52524484	*rs79909872*	0.013	C	A	6.243	2.504-15.564	3.931	8.47×10^{-5}
11	120934552	*rs188394473*	0.012	T	C	6.287	2.513-15.729	3.930	8.48×10^{-5}
1	160052096	*rs56211999*	0.037	C	T	2.710	1.646-4.461	3.921	8.83×10^{-5}
6	9701648	*rs875198*	0.032	T	C	3.156	1.776-5.609	3.918	8.94×10^{-5}
15	65703625	*rs34355056*	0.039	C	G	0.358	0.214-0.599	-3.915	9.04×10^{-5}
10	15648104	*rs2282384*	0.211	G	A	1.624	1.273-2.071	3.907	9.36×10^{-5}
20	36216055	*rs62206398*	0.088	G	A	1.982	1.406-2.794	3.906	9.37×10^{-5}

11	100473583	*rs74571677*	0.072	G	A	0.475	0.327-0.691	-3.904	9.45×10^{-5}
4	134983701	*rs62318231*	0.054	C	T	2.350	1.529-3.614	3.895	9.84×10^{-5}
Significant 48 SNPs in NGT vs T2D									
15	46131802	*rs76400217*	0.030	C	T	8.047	3.513-18.434	4.932	8.12×10^{-7}
12	3923572	*rs3741933*	0.101	A	G	3.143	1.921-5.143	4.561	5.10×10^{-6}
2	59119558	*rs42828*	0.029	G	A	6.822	2.962-15.713	4.512	6.41×10^{-6}
2	233824623	*rs4499416*	0.484	C	T	0.500	0.366-0.683	-4.363	1.28×10^{-5}
12	33579218	*rs185570590*	0.017	G	C	10.379	3.598-29.936	4.331	1.49×10^{-5}
10	71518278	*rs4746907*	0.076	C	A	3.126	1.856-5.264	4.288	1.80×10^{-5}
2	3794696	*rs357975*	0.225	T	C	2.143	1.512-3.037	4.284	1.84×10^{-5}
11	95880984	*rs1939472*	0.202	C	T	2.195	1.503-3.150	4.272	1.94×10^{-5}
11	87940844	*rs599020*	0.150	T	C	2.376	1.584-3.565	4.182	2.89×10^{-5}
11	4870269	*rs12417164*	0.040	A	T	4.698	2.270-9.720	4.172	3.03×10^{-5}
20	41434002	*rs77115494*	0.030	G	A	5.287	2.406-11.617	4.147	3.37×10^{-5}
5	57790085	*rs145755935*	0.015	G	A	9.136	3.205-26.041	4.141	3.46×10^{-5}
2	205269394	*rs143531619*	0.012	T	C	14.120	4.029-49.488	4.139	3.49×10^{-5}
2	59127207	*rs79862664*	0.025	A	G	6.523	2.683-15.858	4.139	3.49×10^{-5}
4	10456414	*rs78432726*	0.026	G	A	0.081	0.025-0.266	-4.136	3.53×10^{-5}
11	114393636	*rs116829295*	0.012	C	T	12.753	3.811-42.670	4.133	3.58×10^{-5}
2	233823850	*rs2176309*	0.179	T	C	0.416	0.274-0.631	-4.127	3.67×10^{-5}
6	166003671	*rs3008004*	0.466	T	C	0.528	0.389-0.715	-4.126	3.69×10^{-5}
14	43317698	*rs61991294*	0.019	C	G	8.073	2.958-22.031	4.079	4.52×10^{-5}
9	7164502	*rs1556099*	0.356	C	A	1.911	1.399-2.609	4.072	4.65×10^{-5}
17	29745981	*rs7406402*	0.286	A	G	0.497	0.355-0.696	-4.068	4.75×10^{-5}
3	194633915	*rs117948724*	0.035	C	T	4.546	2.190-9.440	4.064	4.83×10^{-5}
14	70357182	*rs17107388*	0.077	T	C	3.071	1.781-5.295	4.038	5.39×10^{-5}
10	118219985	*rs12257773*	0.127	C	T	2.423	1.574-3.729	4.024	5.73×10^{-5}
9	36746022	*rs16933516*	0.121	C	T	2.526	1.606-3.973	4.012	6.01×10^{-5}
19	48689859	*rs56345112*	0.073	G	A	3.089	1.780-5.359	4.012	6.02×10^{-5}
6	87498695	*rs2026899*	0.329	G	A	1.888	1.382-2.579	3.994	6.48×10^{-5}
15	26393518	*rs74375088*	0.117	G	A	0.357	0.215-0.593	-3.987	6.69×10^{-5}
2	3745967	*rs62106593*	0.096	C	T	2.713	1.660-4.431	3.987	6.70×10^{-5}
2	59120824	*rs42826*	0.042	T	G	4.128	2.055-8.292	3.985	6.74×10^{-5}
14	74241850	*rs62006091*	0.281	T	G	0.496	0.351-0.701	-3.977	6.97×10^{-5}
1	170084638	*rs117428531*	0.109	A	G	0.347	0.206-0.586	-3.965	7.35×10^{-5}
5	4205074	*rs7734004*	0.301	T	G	0.499	0.354-0.704	-3.959	7.51×10^{-5}
10	71523490	*rs75230499*	0.068	C	A	3.134	1.779-5.521	3.956	7.62×10^{-5}
4	107218105	*rs4956174*	0.034	T	C	0.120	0.042-0.345	-3.943	8.04×10^{-5}
14	100653730	*rs8007801*	0.274	G	C	1.978	1.408-2.778	3.938	8.23×10^{-5}
2	120662756	*rs1588471*	0.026	G	A	5.951	2.447-14.471	3.935	8.32×10^{-5}

17	39493493	*rs76408729*	0.023	C	T	0.099	0.031-0.313	-3.933	8.40×10^{-5}
4	107269031	*rs147386651*	0.045	G	A	0.170	0.070-0.411	-3.933	8.41×10^{-5}
10	125678755	*rs1878618*	0.282	A	G	1.952	1.398-2.727	3.927	8.62×10^{-5}
6	98077320	*rs9372489*	0.219	A	G	1.990	1.411-2.806	3.926	8.64×10^{-5}
5	92149252	*rs1429054*	0.144	C	T	2.291	1.514-3.465	3.925	8.66×10^{-5}
3	173789802	*rs79083143*	0.076	T	C	0.278	0.147-0.528	-3.921	8.82×10^{-5}
5	57156325	*rs4699913*	0.046	T	G	3.855	1.960-7.580	3.913	9.13×10^{-5}
21	40066296	*rs192198209*	0.011	A	G	0.002	0.000-0.040	-3.904	9.45×10^{-5}
17	48725443	*rs144548329*	0.015	A	G	7.901	2.796-22.331	3.901	9.59×10^{-5}
2	112120356	*rs190262259*	0.063	G	A	3.180	1.777-5.689	3.898	9.70×10^{-5}

[a] Chromosome; [b] base pair position; [c] hg19, dbSNP150 version; [d] minor allele frequency; [e] reference allele; [f] alternative allele; [g] odd ratio; [h] confidence interval.

Table S4. Functional annotation of the 86 SNPs associated with T2D status.

Chr	rsID	Alt	CADD [a]	DANN [b]	Protein binding [c]	RDB Score [d]	Gene function	Nearest gene	Phenotypes [e]
1	*rs2993482*	G	**15.260**	0.681	EZH2 [124]	5	intron	*PRDM16*	Obesity [125-129], Hypertension [130,131], Cardiovascular [132], Insulin signaling [133-136]
1	*rs77859272*	T	1.639	0.685	MAFG [137]	5	intron	*DAB1*	Obesity [138], Hypertension [139], Insulin signaling [140-142]
1	*rs1857814*	A	**12.810**	**0.899**	FOS [143], POLR2A	4	intron	*ADGRL2*	-
1	*rs56211999*	T	**14.440**	**0.875**	EZH2	4	intron	*KCNJ9*	-
1	*rs117428531*	G	6.425	0.741	REST, GATA3 [144,145]	4	intron	*KIFAP3*	T2D, Cardiovascular [146]
1	*rs4539115*	G	1.951	0.409	-	5	downstream	*LOC101929184*	-
2	*rs62106593*	T	0.217	0.393	-	5	intron	*ALLC*	-
2	*rs357975*	C	2.681	0.697	-	5	intron	*DCDC2C*	-
2	*rs11675993*	C	2.597	0.363	SPL1	4	intron	*KCNS3*	-
2	*rs42828*	A	4.845	0.717	ZNF24	5	intron	*LINCO1122*	-
2	*rs42826*	G	9.325	0.688	-	5	intron	*LINCO1122*	-
2	*rs79862664*	G	5.022	0.625	-	6	intron	*LINCO1122*	-
2	*rs190262259*	A	1.075	0.338	-	5	intron	*LINC00152*	-
2	*rs1588471*	A	0.539	0.516	-	7	intron	*PTPN4*	Atherosclerosis [147]
2	*rs62174227*	C	**22.500**	**0.922**	-	4	exon synonymous variant	*SP9*	-
2	*rs143531619*	C	5.841	0.691	-	5	downstream	*PARD3B*	-
2	*rs62187185*	T	0.695	0.582	RFX3	4	upstream	*ACSL3*	T2D [148], Cardiovascular [149], Insulin signaling [150-152]
2	*rs2176309*	C	0.944	0.408	-	5	intron	*NGEF*	-
2	*rs4499416*	T	2.545	0.549	SMAD4 [153,154]	3a	intron	*NGEF*	-
3	*rs76693940*	G	1.640	0.377	ZSCAN29, ZNF589, GTF2A2, MGA, NFRK	4	upstream	*ABI3BP*	Cardiovascular [138]
3	*rs79083143*	C	4.232	0.745	PRDM1 [155]	5	intron	*NLGN1*	Atherosclerosis [156]
4	*rs117948724*	T	5.606	0.744	-	5	downstream	*XXYLT1*	Atherosclerosis [157]
4	*rs78432726*	A	0.072	0.660	POLR2A, GATA1	**2b**	intron	*ZNF518B*	T2D [158]
4	*rs77606380*	T	2.032	0.517	-	5	exon	*RPS7P6*	-
4	*rs4956174*	C	2.171	0.640	-	5	intron	*TBCK*	Insulin signaling [159,160]
4	*rs147386651*	A	0.787	0.377	-	7	UTR-3	*AIMP1*	Insulin signaling [161-164]
4	*rs145233789*	C	0.913	0.469	EMSY	5	downstream	*RBM48P1*	-
4	*rs114228107*	A	0.463	0.375	-	7	downstream	*LOC101927359*	-
4	*rs62318231*	T	1.055	0.223	-	6	downstream	*PABPC4L*	-

5	*rs7734004*	G	0.170	0.181	-	5	downstream	*IRX1*	Obesity [165], Cardiovascular [165,166]
5	*rs4699913*	G	2.020	**0.918**	GATA3 [144,145]	4	upstream	*LOC401188*	-
5	*rs145755935*	A	11.710	**0.855**	-	6	UTR-5	*GAPT*	Insulin signaling [167,168]
5	*rs1429054*	T	1.248	0.457	-	5	upstream	*LDHBP3*	-
6	*rs875198*	C	0.381	0.653	-	7	intron	*OFCC1*	-
6	*rs11967600*	T	1.826	0.335	-	**1f**	downstream	*HLA-C*	T1D [169,170], T2D [171,172], obesity [173]
6	*rs2026899*	A	1.521	0.486	ATF2, ATF7	4	upstream	*HTR1E*	-
6	*rs9372489*	G	7.190	**0.837**	-	5	upstream	*LOC101927314*	-
6	*rs3008004*	C	1.713	0.469	-	7	intron	*PDE10A*	T2D, Obesity [174-176]
6	*rs714189*	C	2.439	0.346	-	5	upstream	*LINC00574*	-
8	*rs143491218*	A	0.173	0.260	CLOCK [177,178]	4	upstream	*SMARCE1P4*	-
8	*rs12682560*	C	5.461	**0.807**	-	6	downstream	*MIR12123*	-
8	*rs76088403*	T	1.791	0.767	NR2C1, ZBTB11, ZNF592		downstream	*C8orf33*	-
9	*rs1556099*	A	1.116	0.336	-	5	intron	*KDM4C*	Obesity [179,180], Insulin signaling [181]
9	*rs16933516*	T	2.470	0.692	REST	4	downstream	*MELK*	Obesity [102,104,182,183], T2D, Insulin signaling [102-104,182,184]
10	*rs10458822*	G	2.445	0.687	-	5	intron	*CAMK1D*	T2D [185], Obesity [186], Cardiovascular [187], Insulin signaling [188]
10	*rs2282384*	A	**14.680**	0.782	-	4	intron	*ITGA8*	Cardiovascular [189,190]
10	*rs4746907*	A	0.463	0.711	-	4	downstream	*RPL5P26*	-
10	*rs75230499*	A	0.997	0.348	SPL1	4	downstream	*RPL5P26*	-
10	*rs12257773*	T	0.594	0.395	CEBPB	3a	intron	*PNLIPRP3*	-
10	*rs1878618*	G	3.877	0.270	-	5	intron	*CPXM2*	Hypertension, Cardiovascular [191,192]
11	*rs12417164*	T	**23.300**	**0.993**	CTCF, HNF4A, RAD21, RXRA	3a	exon	*MMP26*	Cardiovascular [192]
11	*rs7102266*	A	**18.370**	0.797	EZH2, MYNN	3a	downstream	*STK33*	Insulin signaling [193,194]
11	*rs599020*	C	0.075	0.321	-	7	downstream	*CTSC*	T1D [195], Cardiovascular [196], Insulin signaling [197,198]
11	*rs1939472*	T	3.831	0.684	-	7	intron	*MAML2*	-
11	*rs74571677*	A	1.639	0.361	POLR2A, SIN3A, FOXA2	3a	downstream	*RN7SL222P*	-
11	*rs116829295*	T	**16.560**	**0.998**	GLIS1 PRDM6	4	missense	*NXPE1*	-
11	*rs188394473*	C	**18.430**	0.752	CTCF, RAD21, RFX3, ZNF7	4	intron	*TBCEL*	-
12	*rs3741933*	G	6.069	0.737	-	7	intron	*PARP11*	-

12	*rs185570590*	C	**17.770**	**0.951**	-	7	missense	*SYT10*	-
14	*rs61991294*	G	0.024	0.304	-	6	upstream	*YWHAQP1*	-
14	*rs17107388*	C	9.712	**0.835**	CHD4	5	intron	*SMOC1*	T2D [199]
14	*rs62006091*	G	0.906	0.638	CTCF [200-202]	4	intron	*ELMSAN1*	-
14	*rs76610020*	T	0.019	0.751	-	5	upstream	*RNU7-51P*	-
14	*rs8007801*	C	11.450	**0.940**	ZNF770	3a	upstream	*DEGS2*	-
14	*rs116924463*	A	0.222	0.711	NBN, ATF2, CTCF [200-202], MLLT1, RUNX3, EBF1	5	intron	*PPP2R5C*	T2D, Obesity [203,204], Insulin signaling [205]
15	*rs74375088*	A	0.572	0.525	-	5	upstream	*LINC00929*	-
15	*rs12591780*	A	8.613	0.669	-	5	downstream	*LINC02345*	-
15	*rs1704349*	A	1.306	0.691	HAUS2	**1f**	intron	*PLA2G4E*	Obesity [61], Cardiovascular [62]
15	*rs76400217*	T	0.562	0.592	-	5	intron	*LOC105370802*	-
15	*rs143940303*	A	1.361	0.192	SCRT2	5	intron	*LIPC*	T2D, Obesity [206,207], Cardiovascular, Hypertension, Hyperlipidemia [208,209]
15	*rs34355056*	G	11.450	0.526	GLIS2, ZIC2, GLIS1, ZNF423	4	missense	*IGDCC4*	-
15	*rs8042910*	C	1.483	0.574	-	6	intron	*VWA9*	-
15	*rs3743171*	A	9.910	**0.979**	-	5	missense	*SLC24A1*	-
17	*rs77103270*	G	1.166	0.444	GLIS2, GATA2, POLR2A	**2b**	UTR-3	*MPRIP*	T2D, Cardiovascular, Hypertension [210]
17	*rs7406402*	G	3.295	0.773	ZFHX2, SCRT2, NR3C1	4	intron	*RAB11FIP4*	Insulin signaling [211]
17	*rs76408729*	T	3.181	0.498	-	5	downstream	*KRT33A*	-
17	*rs144548329*	G	5.904	0.763	IKZF1	4	intron	*ABCC3*	Obesity [212-214], Insulin signaling [215-217]
17	*rs1940653*	A	8.236	0.695	-	6	intron	*AIDAP3*	-
18	*rs79909872*	A	1.248	0.468	-	7	intron	*RAB27B*	Obesity [218], Insulin signaling [219]
18	*rs8090051*	A	0.279	0.278	-	6	intron	*NETO1*	Atherosclerosis [220]
19	*rs72620558*	C	2.110	0.357	USF1, ZNF24, MITF	3a	intron	*CACNA1A*	Obesity [220,221], Hypertension, Cardiovascular [222]
19	*rs56345112*	A	3.617	0.663	BORCS8, MEF2B, CTCF, POLR2A, EBF1	**2b**	intron	*C19orf68*	-
20	*rs78326465*	A	0.697	0.543	-	5	intron	*BPIFA2*	T2D [223]
20	*rs62206398*	A	8.696	**0.891**	-	7	downstream	*GLRXP1*	-
20	*rs77115494*	A	0.665	0.381	-	5	intron	*PTPRT*	T2D, Obesity [224]
21	*rs192198209*	G	0.314	0.373	EZH2	7	intron	*ERG*	Obesity [225], Hypertension,

	Cardiovascular, Hyperlipidemia [226], Insulin signaling [227-229]

[a] CADD PHRED score > 12.37 is bolded as suggested deleterious variant; [b] The DANN score ranges from 0 to 1, with values closer to 1 indicating a higher likelihood of being deleterious; [c] Protein-binding site of transcriptome; [d] RDB rank score ranges from 1 to 6, and a lower number indicates a more deleterious; [e] The association has been investigated in the literature with insulin signaling and T2D-related eight phenotypes; T1D, T2D, obesity, hypertension, hyperlipidemia, cardiovascular, atherosclerosis and insulin signaling.

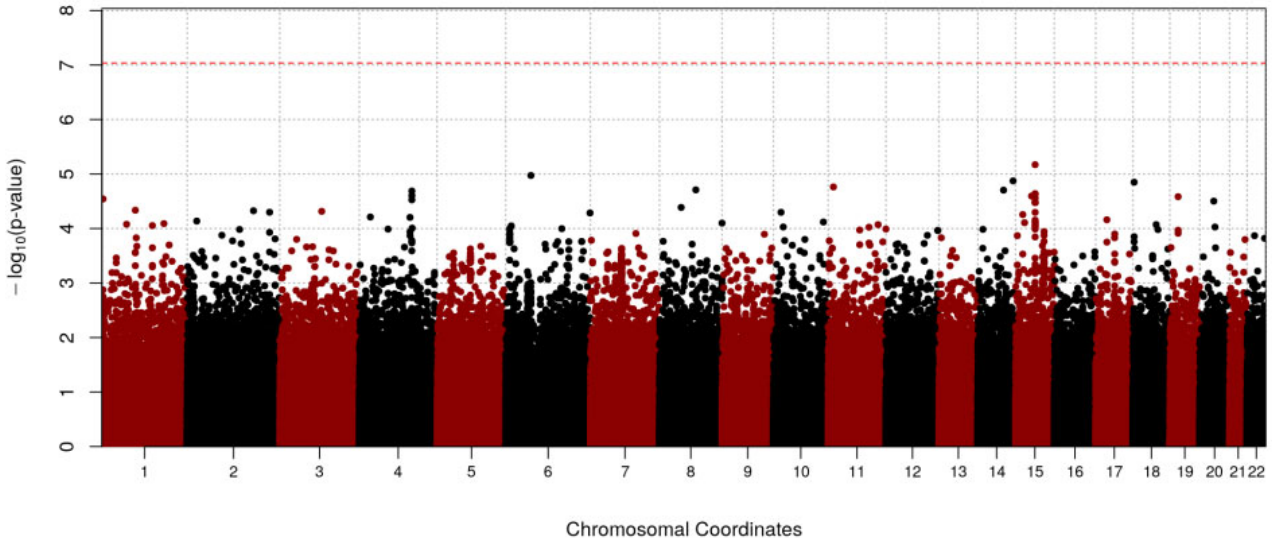

Figure S1. Manhattan plot of the metabolite-adjusted GWAS for the NGT vs PD comparison. The black and red colors are used alternately to distinguish between adjacent chromosomes.

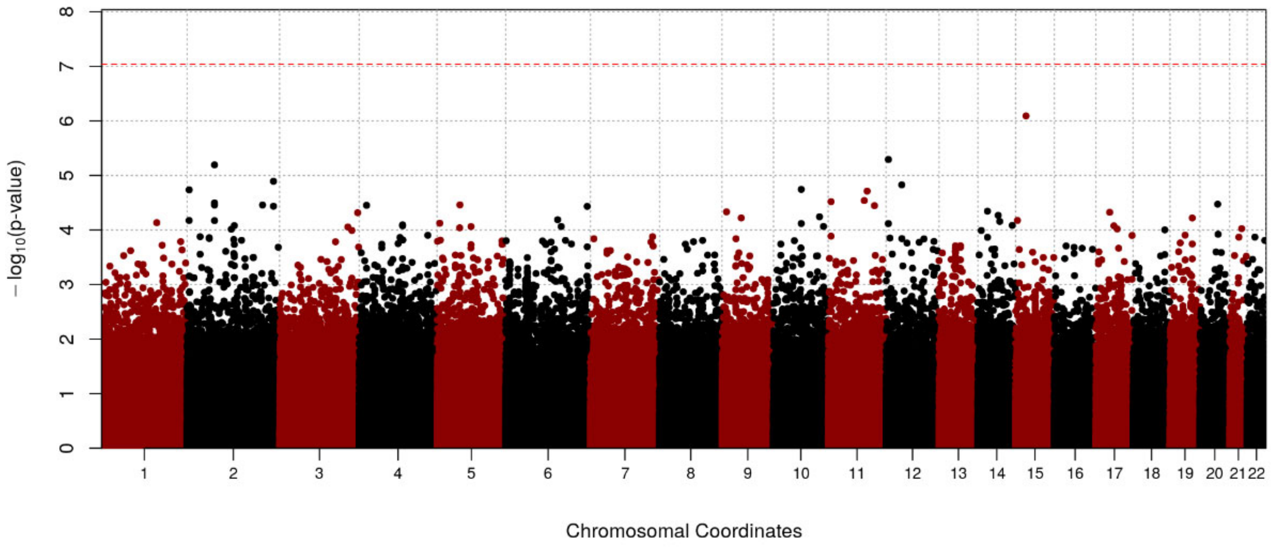

Figure S2. Manhattan plot of the metabolite-adjusted GWAS for the NGT vs T2D comparison. The black and red colors are used alternately to distinguish between adjacent chromosomes.
